# Eosinophilic Fasciitis With Overlap Connective Tissue Disease: A Diagnostic Challenge in Progressive Skin Fibrosis

**DOI:** 10.7759/cureus.114882

**Published:** 2026-08-20

**Authors:** Thanda Aung, Diana Tran

**Affiliations:** 1 Rheumatology, University of California Los Angeles, David Geffen School of Medicine, Los Angeles, USA

**Keywords:** connective tissue disease, eosinophilic fasciitis, myositis, overlap syndrome, sjögren's syndrome, skin fibrosis

## Abstract

Eosinophilic fasciitis (EF) is a rare connective tissue disorder characterized by inflammation and fibrosis of the deep fascia and adjacent subcutaneous tissue. Because its clinical presentation may resemble systemic sclerosis (SSc), diagnosis can be delayed, particularly in patients with an established autoimmune disease. We report a 48-year-old woman with undifferentiated connective tissue disease (UCTD), inflammatory arthritis, and Sjögren’s disease overlap syndrome who developed progressive skin thickening and loss of range of motion despite treatment for inflammatory arthritis. Although EF typically spares the fingers, this patient developed marked finger and hand tightness with inability to make a fist, resulting in an atypical sclerodactyly-like presentation that initially raised concern for SSc. However, the absence of Raynaud phenomenon, normal nailfold capillaroscopy, repeatedly negative SSc-specific antibodies, historical peripheral eosinophilia, and magnetic resonance imaging (MRI) demonstrating fascial thickening and enhancement supported a diagnosis of EF. A full-thickness skin-to-fascia biopsy was deferred because of prior glucocorticoid and immunosuppressive exposure and concern for impaired wound healing. The patient was treated with high-dose prednisone followed by a taper, together with mycophenolate and tocilizumab. Over the subsequent six months, she had improved hand closure, grip strength, and mobility, with stable skin findings at 18 months. This case highlights that EF can occasionally involve the fingers and mimic SSc, and that reviewing the overall clinical phenotype and historical laboratory data is important when the presentation is atypical.

## Introduction

Eosinophilic fasciitis (EF), or Shulman syndrome, is an uncommon immune-mediated sclerosing disorder characterized by inflammation and fibrosis of the fascia and adjacent subcutaneous tissues [1]. Patients typically develop symmetric swelling, induration, and tightening of the extremities, often with restricted joint mobility. Because these findings may resemble systemic sclerosis (SSc), diagnosis can be challenging and treatment may be delayed.

EF and SSc have important clinical differences. EF generally affects the extremities and trunk while sparing the face and fingers, and lacks the characteristic microvascular abnormalities of SSc, including Raynaud phenomenon and abnormal nailfold capillaroscopy [2,3]. Peripheral eosinophilia is common early in EF but may be transient. MRI can demonstrate fascial edema, thickening, and enhancement and may be particularly useful when the diagnosis is uncertain [3,4]. A recent systematic review and meta-analysis of 597 patients found that 13.3% had a coexisting autoimmune disease, although the specific patterns of overlap remain incompletely defined [5]. The prominent finger involvement in our patient was therefore atypical for EF and contributed to the initial concern for SSc.

We report a case of EF in a patient with undifferentiated connective tissue disease (UCTD), inflammatory arthritis, and Sjögren’s disease overlap syndrome whose progressive skin tightening, including marked finger involvement, was initially attributed to possible SSc. The case illustrates the importance of reassessing the diagnosis when the clinical course does not fit the presumed disease phenotype.

## Case presentation

A 48-year-old woman with UCTD, inflammatory arthritis, and Sjögren’s disease overlap syndrome presented in May 2025 for a second opinion evaluation of progressive skin thickening and loss of range of motion involving the hands and extremities.

She initially developed inflammatory joint symptoms, a photosensitive facial rash, and sicca symptoms in July 2023 and established rheumatology care in September 2023. Based on her clinical presentation, she was initially diagnosed with UCTD with inflammatory arthritis and co-existing Sjögren’s disease. Initial serologic testing showed an antinuclear antibody (ANA) titer of 1:640 with a speckled pattern and a positive Sjögren's syndrome-related antigen A (SSA) antibody (129; reference <20). Double-stranded DNA (dsDNA), Smith/ribonucleoprotein (RNP), SSB, Scl-70, and RNA polymerase III antibodies were negative. Her inflammatory arthritis was treated with a prednisone taper, with a maximum dose of 60 mg daily, as well as methotrexate and hydroxychloroquine, with improvement in joint pain and swelling.

**Figure 1:**
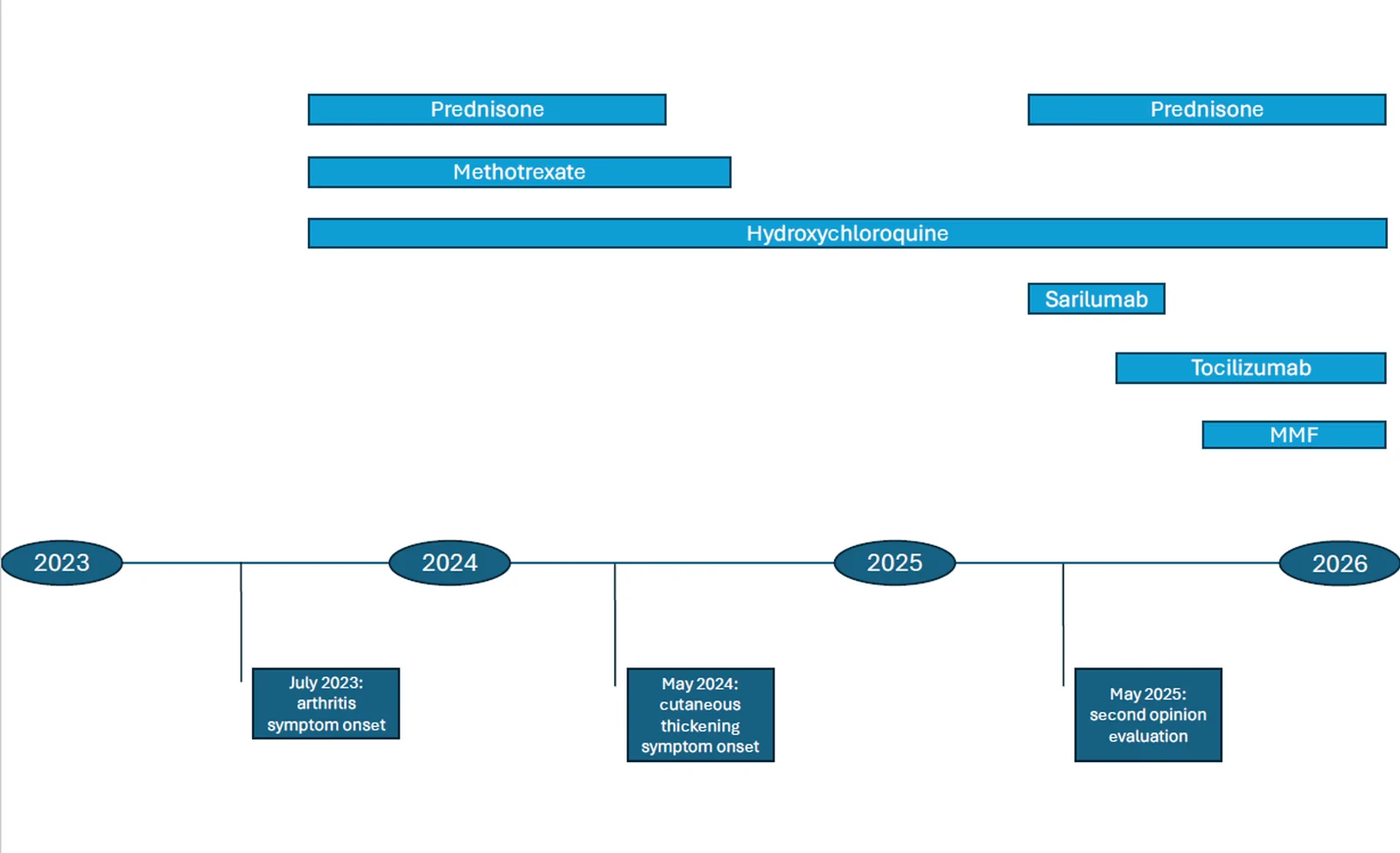
Timeline of Treatment MMF: mycophenolate mofetil

In May 2024, despite improvement in her inflammatory arthritis, she began to develop progressive stiffness, tightness, and thickening of the skin of the proximal and distal upper and lower extremities. She gradually lost the ability to make a fist and had increasingly restrictive hand mobility because of the skin tightness. The new cutaneous findings raised concern for an evolving overlap with SSc. Over the following year, additional immunomodulatory therapies were used, including tofacitinib and sarilumab, with limited improvement in the progressive skin disease. Hydroxychloroquine was continued. A treatment timeline is shown in Figure 1.

**Figure 2:**
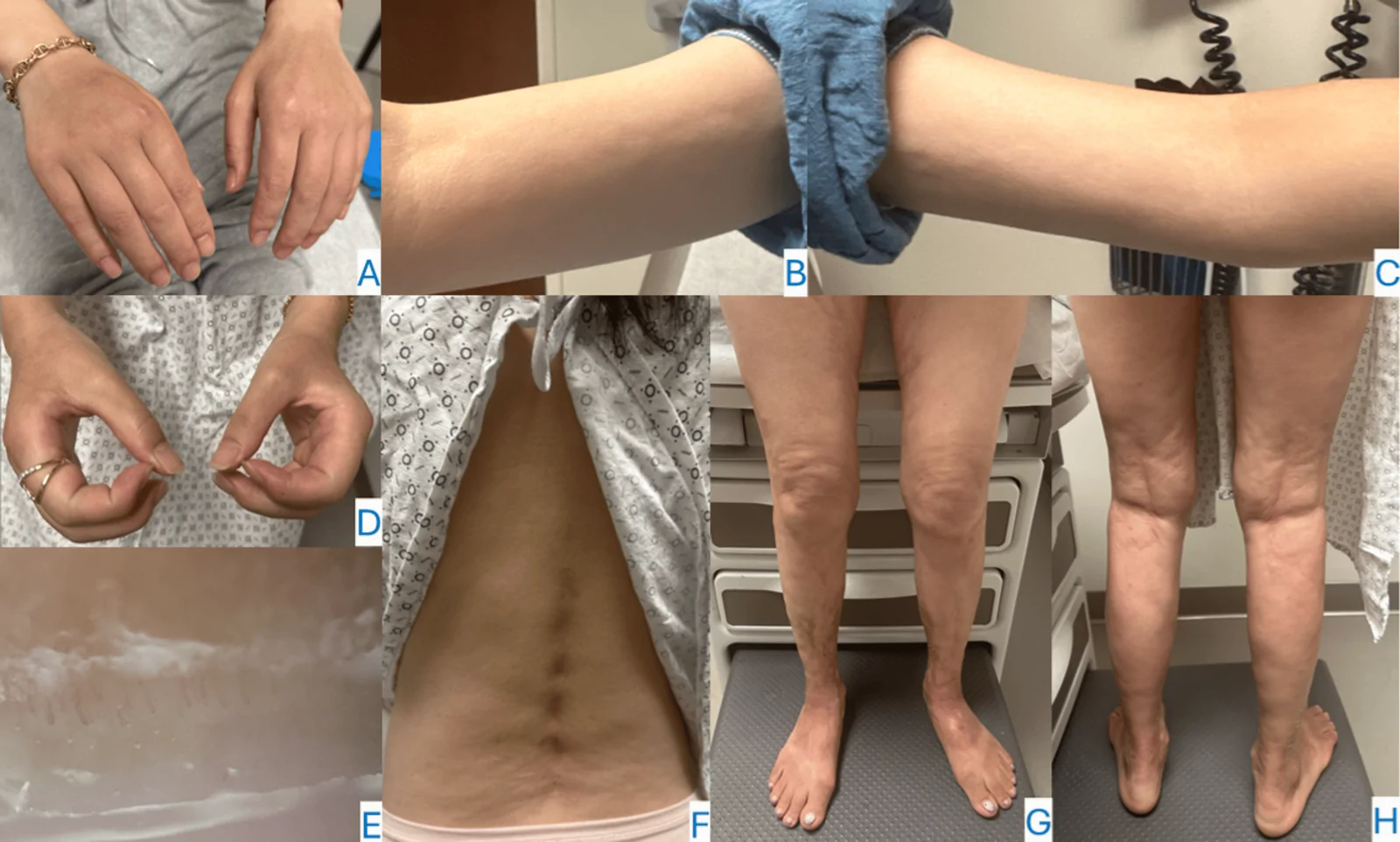
Physical Exam A)-D) Upper extremities with sclerodactyly E) Normal nailfold capillaroscopy of left 4th finger F) Dimpling of back along spine G)-H) Lower extremities with groove sign

At the May 2025 second-opinion evaluation, she reported sicca symptoms, myalgias, and arthralgias but denied Raynaud phenomenon, gastroesophageal reflux, or dysphagia. Examination showed sclerodactyly-like skin thickening distal to the elbows and knees, skin dimpling of the lower back, and traction along the vasculature of the posterior thighs (Figure 2). There was no active synovitis, telangiectasia, or calcinosis. Nailfold capillaroscopy was normal.

Repeat laboratory testing showed ANA 1:1280, dsDNA antibody 1:160, and SSA antibody 156, with negative Scl-70 and RNA polymerase III antibodies and a centromere antibody level below the laboratory cutoff. Complement testing showed low C3 of 79 mg/dL and normal C4 of 12 mg/dL. ESR was 6 mm/hr and CRP was <0.3 mg/dL. The absolute eosinophil count at the time of evaluation was 330/µL. Retrospective review of previous laboratory testing identified an absolute eosinophil count of 1,410/µL (17.2%) around the onset of the cutaneous disease (Table 1).

**Table 1:** Key Laboratory Values ANA: Antinuclear antibody; dsDNA: Anti-double-stranded DNA; SSA: Sjögren's syndrome-related antigen A; Scl-70: Anti-topoisomerase I; RNA Pol III: Anti-RNA polymerase III; AEC: Absolute eosinophil count; ESR: erythrocyte sedimentation rate; CRP: C-reactive protein; C3: complement component 3; C4: complement component 4

Test	Patient Value	Reference Range
ANA	1:1280	<1:80
dsDNA (Crithidia) titer	1:160	<1:10
SSA Antibody	156	<20
Scl-70 Antibody	2	<20
RNA Pol III Antibody	Negative	Negative
Centromere Antibody	<1.0	<1.0
AEC (pre-treatment)	1410/µL	<500/µL
AEC (post-treatment)	330/µL	<500/µL
ESR	6	≤25 mm/hr
CRP	<0.3	<0.3 mg/dL
C3	79	86-175 mg/dL
C4	12	10-40 mg/dL

Although SSc remained an initial consideration because of skin thickening and hand involvement, the absence of Raynaud phenomenon, normal nailfold capillaroscopy, and repeatedly negative SSc-specific antibodies argued against the diagnosis. Systemic lupus erythematosus (SLE) was also considered because of a later positive dsDNA antibody and low C3; however, she did not have oral or nasal ulcers, alopecia, characteristic cutaneous manifestations, renal involvement, or cytopenias to support a diagnosis of SLE.

MRI of the humerus demonstrated diffuse muscle edema with significant thickening and enhancement of the fascia, with mild associated myositis. The MRI images were performed at an outside facility and were not available for independent review for this case report. In the context of the clinical findings and historical eosinophilia, the MRI findings supported a diagnosis of EF. A full-thickness skin-to-fascia biopsy was considered but ultimately deferred because the patient had been receiving multiple immunosuppressive therapies and glucocorticoids, raising concern for poor wound healing and infection, as well as potentially reduced diagnostic yield.

The patient was subsequently treated with prednisone 40 mg daily for two weeks, followed by a taper of 5 mg every two weeks, together with mycophenolate and tocilizumab. Over the following six months, she had substantial improvement in extremity flexibility, regained the ability to make a fist, and reported improved grip strength and walking ability. Functionally, she progressed from having difficulty walking and performing daily activities to walking independently without assistance and being better able to care for her two younger children. At 18-month follow-up, the cutaneous thickening remained stable without further functional decline.

## Discussion

EF can closely resemble SSc, making the distinction particularly challenging in a patient with established autoimmune diseases. The distinction was even more difficult in our patient because of prominent finger involvement. Although EF classically spares the fingers, she developed marked tightening of the fingers with inability to make a fist, creating a sclerodactyly-like phenotype that initially suggested SSc. The absence of Raynaud phenomenon and nailfold abnormalities, together with repeatedly negative SSc-specific antibodies, argued against SSc. Historical eosinophilia and MRI evidence of fascial inflammation provided additional clues. Importantly, the eosinophilia was present early in the course but had resolved by the time of second specialist evaluation, emphasizing that a normal eosinophil count later in the disease does not exclude EF from the differential diagnosis [2,3].

The association between EF and other autoimmune diseases is well described. A 2025 systematic review and meta-analysis of 597 reported patients found a coexisting autoimmune disease in 13.3% of cases [5]. Thus, the presence of inflammatory arthritis and Sjögren’s disease should not have excluded EF, although it contributed to the initial diagnostic uncertainty in this case.

MRI can be helpful when EF is suspected because fascial edema, thickening, and enhancement are characteristic findings [3,4]. Although MRI demonstrated mild associated myositis, MRI findings of muscle edema are nonspecific, and the comprehensive myositis antibody panel was negative, making a primary inflammatory myopathy less likely. A full-thickness biopsy extending from the skin through the fascia remains an important diagnostic test. In this patient, biopsy was considered but ultimately deferred because she had been receiving multiple immunosuppressive therapies and glucocorticoids, raising concern for poor wound healing and infection, as well as potentially reduced diagnostic yield. The lack of histopathologic confirmation is an important limitation of this case. Nevertheless, the combination of the clinical phenotype, historical eosinophilia, MRI findings, and absence of typical SSc vascular features supported the diagnosis.

Treatment of EF is not standardized, and evidence is largely derived from case series and case reports. Systemic glucocorticoids remain the mainstay of treatment, particularly during the inflammatory phase, with methotrexate or mycophenolate commonly used as steroid-sparing therapy [3,6-8]. In a series of 34 patients, treatment delay of more than six months was associated with poorer outcomes, supporting early recognition and treatment [7].

Our patient's treatment history also illustrates the difficulty of distinguishing persistent disease activity from an incorrect disease phenotype. Her inflammatory arthritis improved with initial therapy, but the progressive skin and fascial process continued despite subsequent disease-modifying anti-rheumatic drug (DMARD) and biologic therapy. Once EF was recognized, treatment with high-dose prednisone followed by a structured taper, together with mycophenolate and tocilizumab, was associated with improvement in hand closure, grip strength, and mobility. The functional improvement was clinically meaningful: she went from having difficulty walking and performing daily activities to walking independently without assistance and being better able to care for her two younger children. Biologic therapies such as rituximab and tocilizumab have been reported for refractory EF, but the available evidence remains limited to small series and case reports [9,10]. Because several therapies were administered together in our patient, the improvement cannot be attributed to tocilizumab or any individual treatment. Rather, the clinical response supports the importance of recognizing the underlying fascial disease and initiating an EF-directed treatment strategy.

This case emphasizes that EF should remain in the differential diagnosis of progressive skin tightening, including in patients with established connective tissue disease. Historical eosinophilia, absence of Raynaud phenomenon, normal nailfold capillaroscopy, and MRI evidence of fascial inflammation can help distinguish EF from SSc. Early recognition is important because persistent fascial inflammation can progress to irreversible fibrosis, contractures, and functional impairment.

## Conclusions

EF should be considered in patients with progressive skin tightening and functional limitation when typical vascular features of SSc are absent. Historical eosinophilia and MRI evidence of fascial inflammation may be particularly useful when eosinophilia is no longer present at the time of evaluation. In patients with an established autoimmune disease, reassessment of the diagnosis is warranted when the clinical phenotype and treatment response do not fit the presumed disease. Greater awareness of the distinguishing features of EF may facilitate earlier diagnosis and optimize patient care.
